# Ambivalent professional identity of early remedial medical students from Generation Z: a qualitative study

**DOI:** 10.1186/s12909-022-03583-5

**Published:** 2022-06-27

**Authors:** Mikio Hayashi, Yusuke Karouji, Katsumi Nishiya

**Affiliations:** Center for Medical Education, Kansai Medical University, 2-5-1 Shinmachi, Osaka, Japan

**Keywords:** Remediation, Professional identity, Generation Z, Undergraduate, Qualitative study

## Abstract

**Background:**

Supporting professional identity development in medical students undergoing remediation in the first few years of their studies is an important topic. However, there is a lack of research on developing an effective and individualised process for successful remediation that targets learner identities. This study examined the identities of Generation Z remedial medical students through the lens of professional identity formation, focusing on the difficulties they faced and the support they sought.

**Methods:**

An exploratory qualitative case study was conducted within a constructivist paradigm. Twenty-two medical students (14 males and 8 females) who had experienced remediation in their first few years of medical university participated in this study. All participants were members of Generation Z. Qualitative data were collected through face-to-face, semi-structured interviews and analysed using thematic analysis.

**Results:**

Medical students undergoing remediation in the first few years experienced resistance to the medical profession and conflict due to the gap between the ideal and the reality they experienced after entering medical university. Students’ professional identities were closely intertwined with their pre-university identities; this affected the process of professional identity formation after entering medical university. They preferred assurances of confidentiality as a prerequisite and immediately sought advice through social networks to support their professional identity development.

**Conclusions:**

When planning professional identity development support for Generation Z medical students undergoing remediation in the first few years, it is necessary to carefully select integrative interaction methods, focus on the context of individual learners, and collaboratively discuss specific responses between students and faculty. The results of this study could be useful to faculty in developing support systems for future remedial medical students that focuses on professional identity development and mentoring of remedial medical students.

## Background

Supporting the professional identity development of medical students undergoing remediation in the early years of medical university is an issue in medical programmes. Early identification and support are important because new medical students tend to struggle with knowledge and skill gaps, ways of thinking, and approaches to learning [1, 2]. In addition, early failure in medical university is a risk factor for later professional misconduct [3–6]. Meanwhile, performance on the National Board of Medical Examiners’ exams early in the curriculum seems to be a promising tool for identifying struggling learners, and such early identification may allow for increased opportunities for corrective action for these learners [7]. It is also useful for identifying learner obstacles and developing appropriately detailed remediation strategies [8, 9]. The increasing diversity of medical students highlights the importance of supporting remedial students from multiple perspectives, focusing on the learner’s context [10, 11]. However, most research on academic remediation in medical education has focused on retaking failed exams, and there is little evidence to determine which students need what kind of help, and what kind of remediation would be most useful for each learner [12].

In addition, the majority of medical students nowadays belong to Generation Z, defined as those born between 1995 and 2012. This generation has been shaped by the widespread growth of the internet, use of smartphones, and the rise of social media, in which distinctive characteristics include social inclusivity and high expectations regarding the immediate access to and delivery of information [13]. It has been also noted that Generation Z has limited face-to-face interactions, leading to increased feelings of loneliness [14–16]. Identity support for medical students in this generation is likely to be more complex because it requires an understanding of their unique characteristics [13]. This study focused on the identities of Generation Z medical students who experienced remediation early in their university careers and explored the difficulties they faced and the support they sought.

In providing support programmes for medical students, medical universities need to consider effective additional support and supplemental instruction rather than focusing solely on improving students’ performance to pass exams or assessments [17]. Intervention studies with medical students have demonstrated the effectiveness of a learner-centred approach [3, 18] and indicated that cognitive and emotional considerations are related to long-term programme effectiveness [11]. In addition, collaborative learning with peers and personal support can be effective for medical students with support needs, as long as peers maintain confidentiality and respect [4, 19]. A recent Best Evidence Medical Education (BEME) review detailed interventions for medical students with academic difficulties. However, the authors of the BEME review noted that very few interventions targeted learner identity, and they suggested that key elements of professional identify formation including guided reflection, use of personal narratives, and role modelling might make a difference for learners who do not feel that they belong to their discipline [18].

Professional identity formation has been defined as an adaptive developmental process that happens simultaneously at two levels: (1) at the level of the individual, which involves the psychological development of the person, and (2) at the collective level, which involves the socialization of the person into appropriate roles and forms of participation in the community [20, 21]. Using professional identity formation as a theoretical framework, we considered that remedial students’ experiences may point to underlying issues that affect not only the content of learners’ values and attitudes, but also how individuals structure those values and attitudes [22]. In addition, fostering remedial medical students’ insight into the process of professional identity formation may encourage them to be more proactive in forming a professional identity aligned with their values [20, 22, 23].

Therefore, the purpose of this study was to explore the professional identity of Generation Z medical students with academic difficulties who experienced early remediation at a medical university in Japan, focusing on professional identity issues and the support they sought. This study aims to contribute to the creation of support strategies for future remedial medical students that focus on professional identity development.

## Methods

### Study design

We followed the recommendations of the Standards for Reporting Qualitative Research (SRQR) [24] and used an exploratory qualitative case study methodology within a constructivist paradigm [25], which considers multiple constructed realities that can be studied holistically. From a constructivist perspective, research outcomes will never be separate from the biases, assumptions, and characteristics of the researcher. Therefore, we discussed the collected data and analysis several times during the study, and ensured that multiple views from study participants were presented and that a major oversight did not occur. A case study is an empirical investigation of a current phenomenon that occurs in a real-world context [26], and this approach allowed us to explore the early remediation experiences of the medical students as the participants of this study [27, 28]. Accordingly, we confirmed that we were in a position to collect and analyse data sources, a situation to which the case study within a constructivist paradigm could be applied.

### Setting

This study was conducted at a six-year medical university in Japan with a capacity of 130 students per year. All medical universities in Japan offer six-year programmes. Formal clinical clerkships are usually conducted in the fourth, fifth, and sixth years of study [29]. The average dropout rate at medical universities in Japan has increased over the past 10 years, especially in the early years [30]. At the medical university where this study was conducted, the overall dropout rate was about 4%, and medical students who had done remedial work had to repeat the year. As in other medical universities in Japan, the requirement for promotion was that all courses were completed. Regarding socio-cultural aspects, Japanese learners were classically considered collectivist and quiet, but in recent years it has become apparent that medical students in Japan are not necessarily passive [31].

### Participants

Medical students who had experienced early remediation due to poor academic performance, lack of credits, or poor class attendance, were recruited for this study. Early remediation in this study is the act of facilitating a correction for trainees who started their journey toward becoming a physician but moved off course in the first few years of their studies. All medical students who met these criteria in 2020 at the university where the study was conducted were invited by email. Participation was voluntary. Twenty-two medical students (14 men and 8 women) agreed to participate in the study. Of them, 13 were first-year students, 6 were second-year students, and 3 were third-year students. The average age of the participants was 24 years (range 21–26 years), falling into Generation Z.

### Ethics

The Institutional Review Board of Kansai Medical University approved this study (2020198). Sensitive information revealed during the interviews was kept confidential. Participants were informed of the scope and nature of the study, and they provided written informed consent to participate. Participants were informed that all data would be kept confidential and that they could withdraw their consent at any time.

### Data collection

Semi-structured interviews were conducted to understand students’ perspectives during remediation [32]. Interviews were conducted face-to-face by the first author (MH) between October 2020 and December 2020. Open-ended questions were used to clarify participants’ perspectives on their remedial experiences and how those experiences contributed to their subsequent living and learning environment as a medical student or their interactions with other medical students. The main questions were as follows: ‘Did you have any problems during your remediation that were difficult to discuss with others?’ and ‘What kind of support do you think is needed for remedial students?’ We used the professional identity theoretical framework as a sensitizing framework for designing the interview questions. The early remedial medical students’ narratives elicited through the main questions were expected to provide useful insights into how they viewed their professional identity.

During the interviews, when necessary, the interviewer also asked for background information on the learner’s decision to enter medical university and listened carefully to the reasons behind the interviewee’s responses in the face of academic challenges. Interviews were conducted in Japanese, tape-recorded, and lasted 20–60 min. The recorded data were transcribed verbatim, immediately after each interview and then translated into English by the authors.

### Data analysis

Interview data were analysed using thematic analysis, which involves generative coding and theorising to identify instances of similar concepts in the dataset [33, 34]. Data analysis followed an inductive approach in which emergent conceptual categories and descriptive themes were observed. Although the research question was partly theory-driven, we initially conducted a primary-level thematic analysis to determine themes. After we identified the themes, and following further team discussions, we re-examined the literature to identify the conceptual lens.

MH was formally trained in NVivo11 for Windows (QSR International, Australia), a programme that supports qualitative data analysis. MH performed all coding steps, including reading and rereading transcripts until themes were identified. Themes were categorised into major and minor categories and tabulated using NVivo11 to determine their frequency. MH identified the themes from the qualitative data; the second (YK) and third (KN) authors reviewed the analysis. For each disagreement, the authors discussed and reviewed the data until consensus was reached.

The authors were faculty members at this medical university and were involved in medical student education. Our research team comprised of two faculty members specializing in medicine (MH, KN) and one faculty member specializing in educational psychology (YK). While the medical researchers provided the emic perspective and associated potential biases, the educational psychologist offered the etic perspective. In addition, MH had experienced early remediation as a medical student and shared personal experiences with participants as needed.

## Results

**Fig. 1:**
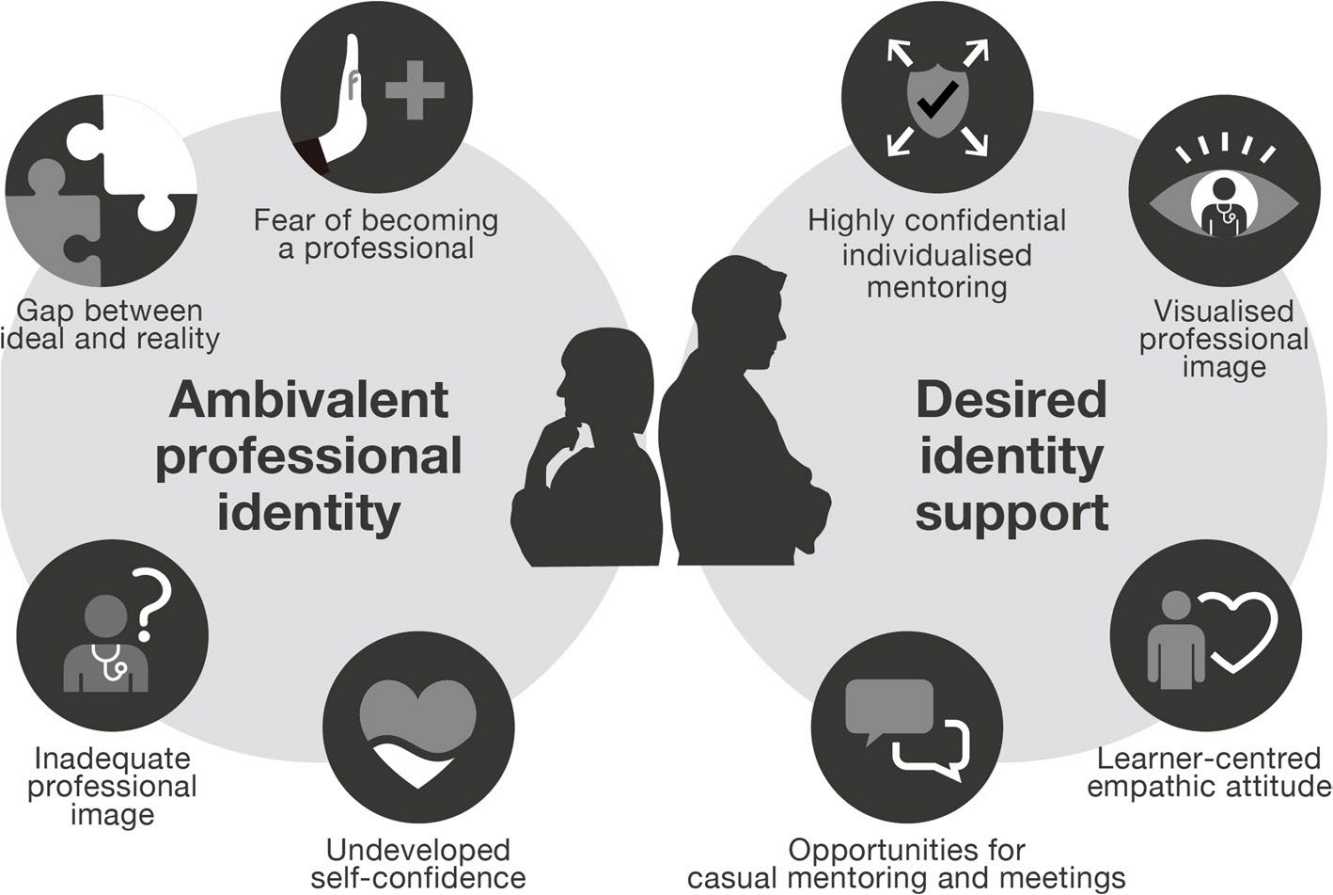
Identified categories. Early remedial medical students from Generation Z experienced a variety of ‘ambivalent professional identities’. Four main categories emerged: fear of becoming a professional, gap between ideal and reality, inadequate professional image, and undeveloped self-confidence. Students also expected ‘desired identity support’, which was divided into four main categories: highly confidential individualised mentoring, visualised professional image, learner-centred empathic attitude, and opportunities for casual mentoring and meetings

Based on the interviews, we found that participants experienced a variety of ‘ambivalent professional identities’, with four main themes identified: fear of becoming a professional, gap between ideal and reality, inadequate professional image, and underdeveloped self-confidence. Participants expected ‘desired identity support’, which was categorised into four main themes: highly confidential individualised mentoring; visualised professional image; learner-centred empathetic attitude; and opportunities for casual mentoring and meetings (Fig. 1).

### Ambivalent professional identity

The concept of ambivalent professional identity encompasses the themes that emerged as a result of deep investigation into how medical students who underwent remediation in the first few years perceived the experience and how their identities fluctuated based on what they perceived as problems before and after remediation. Participants felt resistance toward the medical profession and conflict due to the gap between their prior ideals and the reality that they experienced after entering medical university. In addition, they had underdeveloped self-esteem and felt unable to disclose to their colleagues and parents that they were not motivated to continue their studies because they believed that this discussion would not resolve it. In addition to the university environment, life and family factors were also influential.

#### Fear of becoming a professional

Some participants felt that they had chosen to study medicine because of the expectations of those around them, despite their own tenuous sense of purpose. After entering medical university, they felt trapped in the medical community and had a sense of urgency about the possibility of acculturating:

I didn’t really want to go to medical university, but I didn’t know what else I wanted to do, and my parents wanted me to be a physician; so I compromised on that and enrolled into medical university...Medical university is a very small community and a unique environment. I feel uncomfortable living in this community for the rest of my life, and I am worried that I will go native.

Furthermore, participants were not only aware of differences in university and family environments but also recognised their social stratification through information exchange and interaction with classmates. Additionally, some felt a complex about social inequality:

I felt really jealous of my classmates who grew up in a privileged family environment with high social status because I'm from a rural area...Many of them know the world of medical professionals because of their parents’ influence, so I’m sure their information and ways of dealing with problems will be different from mine.

#### Gap between ideal and reality

Participants experiences a sense of emptiness, because they felt that they were incapable of becoming a physician. They were also burdened with a heavy workload and unable to complete all tasks. They tried too hard to conform to the environment:

I realised that even if I study, I won’t learn anything if I don’t have a sense of involvement...I had a hard time before I entered the medical university, but after I entered the medical university, the amount of study was more than I had imagined, and the hard time accelerated because my own efforts didn’t lead to good grades.

Participants were aware of their unfulfilled desire for recognition, as well as the inadequate recognition they received, because their efforts were not fully appreciated by their parents or the medical university faculty members:

When I was accepted to medical university for the first time, my parents asked me whether I really wanted to go there, and I was discouraged that they didn’t recognise the hard work and effort I had put in. Even after I started going to medical university, I felt that the return on my efforts to learn was too small.

#### Inadequate professional image

Some participants were unable to tell their peers and parents that they had not necessarily entered medical university with a firm desire to become a physician. In addition, they were unable to candidly tell their parents that they were not motivated to continue their studies, because they believed that nothing would be resolved through discussion:

While many of my classmates entered medical university because they wanted to become a physician, I couldn’t tell them the fact that I didn’t...I couldn’t honestly tell my parents about my lack of motivation after entering medical university, and I hid the fact that I wasn’t able to go to university from them.

Participants were unable to fully envision their future as physicians after entering medical university. The reason for remediation was not only the lack of credits and attendance, but also the fact that the remedial students in the first few years could not see their future as a physician, which led to strong resistance to the act of attending classes. They compared their learning environment to that at non-medical universities and were aware of feelings of insufficient free time:

I felt relieved when my seniors in the club told me that I would be able to advance even if I didn’t study a lot...I wanted to live like a real student, just like any other faculty, because I am a student.

#### Undeveloped self-confidence

Participants were overly anxious after entering medical university because they had a limited sense of self-worth. In addition, low resilience caused them to distance themselves from their classmates and delay their progress in the curriculum, even though they understood the requirements:

I knew what I needed to do then, but I couldn’t do it...I felt I wasn’t worth the high tuition at the medical university.

After I entered medical university, my motivation became lower and lower, and I coldly watched my classmates studying...I sometimes went drinking and gambling with my friends, thinking it was the summer vacation of my life.

Although the university offered numerous learning opportunities, the participants indicated that they did not seek out these available opportunities. They believed it was their fault if they did not take advantage of these opportunities and struggled to bridge the gap between feelings and actions. They attempted to resolve these issues by avoiding relationships with classmates and counselling opportunities. Although they felt isolated, they tried to improve their situation on their own:

I told myself that I have some understanding of myself in my own mind and decided not to discuss it with others.

### Desired identity support

Participants sought support for their ambivalent identities and wanted to ensure that their safety was protected and that support was individualised. In addition to formal counselling, they felt that opportunities for interaction, among medical students of different years and with young physicians, as well as immediate advice through social networks, were important for professional identity support. They felt that the job description should be visualised from the student’s perspective before the clerkship.

#### Highly confidential individualised mentoring

Participants were concerned that discussing barriers could lead to the dissemination of personal information and affect their evaluation, and strongly favoured assurances of confidentiality as a condition of support. While they were sceptical of generalised approaches, such as formal mentoring offered to students by faculty, they were very hopeful that individualised approaches tailored to the learning styles of individual students would be offered:

I was worried that the information that I didn’t want to go to medical university would be leaked to the faculty members and that it might affect my grades...I was called by a faculty member to check on my progress when my grades were slipping, but I felt that she was still searching for a way to talk to the students without knowing how to talk to them...I would like the faculty to start by talking to the students in a more familiar way.

Some participants required help and expected teachers to provide them with specific goals and solutions tailored to their learning environments. They also felt that immediate advice and feedback via social media would be useful:

Rather than spending a lot of time explaining things, I’d like them to see why we are struggling...There are only a limited number of opportunities to talk about whether the way of study is really the right way, but I realise that the students themselves need help.

It would be helpful to get an immediate answer via social networking or chat when I need help with something.

#### Visualised professional image

Participants were concerned about the negative impact and uncertainty that the remedial experience itself would have on their future job prospects. Regarding motivation, they indicated that they had no role models nearby. They believed that career planning support would help them improve their motivation:

I have a vague feeling of anxiety about how I should think about my career...I don’t have anyone close to me, but if I had someone like a role model, I might be able to feel more positive about it.

Female medical students in particular were concerned about the possibility of having a ‘career gap’ in the future due to life events such as marriage and childbirth. They continued to worry about becoming a physician after medical university. They also felt it was important to seek faculty advice if required:

Even if I become a physician, there is a possibility that I will have a gap in my career due to childcare after marriage, and when I think about whether I will be able to go back to work, I feel uneasy. I want the faculties to point out directions when I have problems.

#### Learner-centred empathic attitude

Participants wanted to feel safe enough to disclose their concerns about the medical profession to the faculty. They also expected a holistic approach and a tolerant response based on detailed information about their life and family environment, along with their academic performance, to avoid misunderstandings about their personality:

Please don’t call me a problem learner...It is quite difficult for me to reveal my deepest feelings of lack of motivation to become a physician to the faculty, but I try to be as specific as possible because I don’t want my personality to be misunderstood.

Participants did not necessarily expect clear instructions or generous support from the university or faculty. They did, however, expect to be supervised at a reasonable distance and be provided adequate time, owing to their supervisors’ trust in their autonomy.

I don’t think my grades will improve because of the university’s generous support, so I didn’t think I needed any particular generous support...I just want them to wait a little bit until I’m ready to do it on my own, and I feel grateful if they are not too close and not too far away.

#### Opportunities for casual mentoring and meetings

Participants emphasised the importance of a comfortable environment and a sense of familiarity in accepting personal support. They believed that interaction and advice from older students close to their level would be helpful. They also believed that interaction among remedial students would help alleviate psychological distress and hoped that the university would provide such opportunities:

Friends and seniors are easier to talk to, more convincing, and I can complain without hesitation...I think talking to other remedial students makes me feel a little more comfortable because I realise we are all experiencing similar pain. It’s hard to be alone.

In addition, participants wanted to enjoy the efforts of faculty members and other students and felt that encouragement from third parties and classmates would be helpful:

I don’t study for the mentors, but it’s still nice when they tell me how hard I’m working...When I was in a situation where I didn’t know how to study, my classmates made a study schedule and supported me, which was very encouraging.

## Discussion

Participants’ identities were closely intertwined with their identities before entering medical university and influenced the process of later identity development. Although some of the findings of this study are consistent with those of previous studies, such as the importance of learner-centred approaches, individualised contextual responses, and assurances of confidentiality, this study identified trends in the difficulties and desired identity support of Generation Z medical students undergoing remediation in the first few years of their study. As the findings indicate, they experienced many types of ambivalent identities, and faculty members need to listen more to their students and deepen the relationship with them through dialogue. Therefore, remedial medical students in their first few years should not simply be labelled as problem learners. In addition, the expectation of immediate advice and feedback through social networks and increased opportunities for interaction among medical students has deepened students’ desire for support. These unique findings reflect trends among Generation Z medical students, and faculty should work with them to explore mentoring methods using social networks in the future. The results of this study may be useful to faculty members in the development of support systems for future remedial medical students that focus on professional identity development and mentoring in the first few years of study.

The findings suggest that medical students undergoing remediation in the first few years of study have ambivalent identities and expect a holistic approach and tolerant responses. In developmental psychology, one of the pillars of professional identity formation theory, early adulthood is a time of uncertainty and turmoil but also an opportunity, as individuals gradually become more certain of who they want to become [21]. Medical students undergoing remediation in the first few years of study have their unique characteristics and culture from the moment they enter medical university, and the institution expects them to understand and accept the educational goals and cognitive foundations of medical university early on [35]. However, in doing so, they may encounter some obstacles that lead to early remediation. The findings suggest that this may be because of conflicts in existing personal identities upon entering medical university and a desire for recognition by their environment. In other words, although professional identity formation is thought to include socialization into appropriate roles and forms of participation in community work [20, 21], the formation of professional identity of remedial medical students may face obstacles in the transition process from existing identities to socialization.

Participants not only needed immediate support from social media but also interaction with other medical students as tangible support for their professional identity formation. These findings are closely related to previous findings on the usefulness of social media in supporting professional identity formation [21]. More recent approaches to this topic are more balanced, as most medical students use social media for personal and educational reasons, and the current generation of learners is creating online communities that foster a sense of belonging and reduce isolation [36, 37]. However, social media cannot replace face-to-face interaction with peers and mentors. On the other hand, Generation Z is also characterised by a lower sense of responsibility and independence, a desire for face-to-face interaction, and a tendency toward collectivity, although face-to-face social interaction is limited [14, 16]. As the results show, Generation Z medical students expected solutions tailored to their individual learning environment. This result may also be because Generation Z is believed to be more inclined than other generations to not want to be misunderstood by others [13] and therefore want to be accepted for who they are. When planning professional identity support, it is necessary to choose inclusive interaction methods carefully, taking into account the learner’s background context and the generational difference between learners and teachers. While additional research may be required to clarify the general theory of support for Generation Z medical students, we believe that some of the support they desire, as revealed through this qualitative study, may be helpful in further discussions.

Although previous studies have shown that remedial medical students have low self-confidence and tend to harbour negative feelings about learning and their own performance, which exacerbates their problems [1, 3], the findings of this study suggest that underdeveloped self-confidence may be influenced by their concepts of insufficient resilience and moratorium. Meanwhile, the desired support sought by medical students in mentoring is realised not only through individualised approaches but also by ensuring students’ safety. These results could be explained not only by their generation but also by the effect of the collectivist nature of Japanese learners. However, as there are criticisms of generalising learner behaviour [31], when considering support content changes, it is necessary to focus on the individual context of learners and discuss specific responses in collaboration between students and faculty.

This study has some limitations. It was conducted at a single medical university with a group of early-career students; therefore, given the importance of context in professional identity formation, the results may not be transferable. In addition, the small number of participants may indicate that the conceptual framework is based on unique student experiences and may not be generalisable. Further research in other contexts is needed to examine the transferability of these findings. In addition, this study was not longitudinal. Continuing to follow participants during their clinical clerkship would allow us to track the development of their professional identities and provide further insight into how they used their early healing experiences. Finally, although we conducted a thematic analysis because it fit the constructivist approach, other methodological and analytic approaches might reveal different perspectives on the relationship between ambivalent professional identity and desired identity support in remediation.

## Conclusions

This study clarified the difficulties and desired support of Generation Z remedial medical students through the lens of professional identity formation. When planning professional identity development support for these students, integrative interaction methods that focus on the context of the individual learners themselves must be carefully selected, and specific responses should be discussed in collaboration with students and faculty. The details of an ambivalent professional identity and desired identity support derived from this study will be useful to faculty in mentoring remedial medical students. We believe that through a detailed and careful dialogue that takes into account that Generation Z medical students are more inclined than other generations to not want to be misunderstood by others, it may be necessary for faculty to understand the formation of the existing identities of medical students who have experienced remediation and, if necessary, how they can be transferred to the process of socialization. Increasing opportunities for medical students to engage with various types of role models early on in their medical school careers to help them develop their professional image may contribute to professional identity support. In addition to providing direct verbal advice to students who have experienced remediation, it may be useful to provide prompt and detailed advice through various tools, such as social network services, in order to support the professional identity of Generation Z medical students. Further research on medical students’ professional identity development should be conducted in a variety of contexts.

## Data Availability

The datasets generated and/or analysed during the current study are not publicly available in order to protect the originality of our work, so that we can continue evaluating future examinees. However, they are available from the corresponding author on reasonable request.
